# Delirium Audit Project of the Greenwich Older Adult Mental Health Liaison Service

**DOI:** 10.1192/bjo.2024.641

**Published:** 2024-08-01

**Authors:** Anil Ustun

**Affiliations:** Oxleas NHS Foundation Trust, London, United Kingdom

## Abstract

**Aims:**

Delirium poses a significant risk in hospitals, with a prevalence of 20–30%. Queen Elizabeth Hospital conducted an audit focusing on delirium cases referred to the Greenwich Mental Health Liaison Team for Older Adults (GMHLT OA) between January and April 2023.

The audit aimed to assess immediate and medium-term outcomes, identify improvement areas, and propose strategies for optimizing delirium treatment within GMHLT OA.

**Methods:**

Patient referrals received by OAMHLT are meticulously recorded in a logbook. Among the referrals, 39 patients from the target population were identified through a manual review of the documentation. To augment the data collection process, electronic databases were also reviewed to ensure comprehensive data retrieval.

**Results:**

Key Findings:

39 cases audited, predominantly females (62%).

Most affected age group: 71–80 years.

Infective causes (49%) and low mood (30%) were common.

Antipsychotic treatment administered in 56% of cases.

36% required institutionalization post-discharge.

**Conclusion:**

The audit underscores the complexity of delirium care, aligning with epidemiological data. It provides a foundation for targeted improvements to enhance patient outcomes within GMHLT OA. Based on the results the following recommendations and action plan were made:

Implement multifaceted interventions and non-pharmacological approaches.

Strengthen collaboration between departments for diverse referral sources.

Explore regional resource allocation and establishment of care pathways based on local implications.

